# Infections and Immunotherapy in Lung Cancer: A Bad Relationship?

**DOI:** 10.3390/ijms22010042

**Published:** 2020-12-22

**Authors:** Lorenzo Belluomini, Alberto Caldart, Alice Avancini, Alessandra Dodi, Ilaria Trestini, Dzenete Kadrija, Marco Sposito, Daniela Tregnago, Miriam Casali, Silvia Teresa Riva, Giulia Sartori, Jessica Menis, Michele Milella, Sara Pilotto

**Affiliations:** 1 Section of Oncology, Department of Medicine, University of Verona Hospital Trust, 37134 Verona, Italy; lorenzo.belluomini08@gmail.com (L.B.); alberto.caldart@gmail.com (A.C.); alessandra.dodi92@gmail.com (A.D.); ilariatrestini92@gmail.com (I.T.); dzenete.kadrija@gmail.com (D.K.); marcosposito91@gmail.com (M.S.); danielatregnago@libero.it (D.T.); mi.ma.casali@gmail.com (M.C.); silviateresariva@gmail.com (S.T.R.); sartorigmt@gmail.com (G.S.); michele.milella@univr.it (M.M.); 2 Biomedical, Clinical and Experimental Sciences, Department of Medicine, University of Verona Hospital Trust, 37134 Verona, Italy; alice.avancini@univr.it; 3 Department of Surgery, Oncology and Gastroenterology, University of Padova, 35124 Padova, Italy; j16menis@gmail.com; 4 Medical Oncology Department, Istituto Oncologico Veneto IRCCS, 35128 Padova, Italy

**Keywords:** non-small cell lung cancer, antibiotic therapy, microbiota, HIV, antiretroviral therapy, HBV, HCV, COVID-19

## Abstract

Infectious diseases represent a relevant issue in lung cancer patients. Bacterial and viral infections might influence the patients’ prognosis, both directly affecting the immune system and indirectly impairing the outcome of anticancer treatments, mainly immunotherapy. In this analysis, we aimed to review the current evidence in order to clarify the complex correlation between infections and lung cancer. In detail, we mainly explored the potential impact on immunotherapy outcome/safety of (1) bacterial infections, with a detailed focus on antibiotics; and (2) viral infections, discriminating among (a) human immune-deficiency virus (HIV), (b) hepatitis B/C virus (HBV-HCV), and (c) Sars-Cov-2. A series of studies suggested the prognostic impact of antibiotic therapy administration, timing, and exposure ratio in patients treated with immune checkpoint inhibitors, probably through an antibiotic-related microbiota dysbiosis. Although cancer patients with HIV, HBV, and HCV were usually excluded from clinical trials evaluating immunotherapy, some retrospective and prospective trials performed in these patient subgroups reported similar results compared to those described in not-infected patients, with a favorable safety profile. Moreover, patients with thoracic cancers are particularly at risk of COVID-19 severe outcomes and mortality. Few reports speculated about the prognostic implications of anticancer therapy, including immunotherapy, in lung cancer patients with concomitant Sars-Cov-2 infection, showing, to date, inconsistent results. The correlation between infectious diseases and immunotherapy remains to be further explored and clarified in the context of dedicated trials. In clinical practice, the accurate and prompt multidisciplinary management of lung cancer patients with infections should be encouraged in order to select the best treatment options for these patients, avoiding unexpected toxicities, while maintaining the anticancer effect.

## 1. Introduction

The use of immune checkpoint inhibitors (ICIs), both alone or in combination with chemotherapy, has radically changed the treatment algorithm of locally advanced and metastatic non-small cell lung cancer (NSCLC), improving patients’ survival with a favorable safety profile [1].

Nevertheless, ICIs’ efficacy and safety in less-investigated special subgroups of lung cancer patients are still far from being clarified. Indeed, lung cancer patients could suffer from concomitant infections.

With regard to bacterial infections, to which lung cancer patients are particularly susceptible, several studies reported the prognostic implications of antibiotic therapy administration, timing, and cumulative exposure ratio (proportion between days of antibiotic therapy and days of immunotherapy) [2,3].

Patients concurrently affected by lung cancer and viral infections, such as the human immune-deficiency virus (HIV), hepatitis B virus (HBV), and hepatitis C virus (HCV), have usually been excluded from those clinical trials that led to immunotherapy regulatory approval, mainly due to concerns about tolerance, efficacy, and risk of viral reactivation. However, a series of case reports, retrospective, and recent prospective studies are available, suggesting that ICIs seem to be safe and active in people living with HIV (PLWH) and in patients affected by chronic HBV/HCV, without reliable risks of viral reactivation [4,5].

Among viral infections, since the end of 2019, the severe acute respiratory syndrome coronavirus 2 (SARS-CoV-2), which causes the coronavirus disease 2019 (COVID-19), is progressively spreading worldwide [6]. Overall, patients with cancer appeared to be at higher risk of severe events and death compared with non-cancer patients [7,8,9]. Among oncological patients, those affected by lung cancer seem to be particularly vulnerable, with an estimated mortality rate of around 32% [10]. Nevertheless, data exploring the potential correlation between the type and timing of anticancer therapies, including immunotherapy, and COVID-19 outcome are still preliminary.

Herein, we aim to review the available evidence about the use of immunotherapy in lung cancer patients concurrently affected by bacterial or viral infections, including a brief focus on the COVID-19 disease.

## 2. Bacterial Infections in Lung Cancer

Patients affected by NSCLC have a higher risk of experiencing recurrent infections related to the oncological disease itself, to frequently co-occurring factors, such as old age, smoking history, presence of chronic obstructive pulmonary disease (COPD) [11,12], as well as to the immunosuppression related to oncological therapies [13]. The majority of infections involve the respiratory tract, mainly bacterial pneumonia, typically diagnosed with productive cough with fever and/or radiological infiltrates [13,14]. Other frequent infections may involve blood, urinary, or the gastrointestinal tract [15].

According to the etiology, the most commonly administered antibiotics are broad spectrum, mainly β-lactams (+/− inhibitors), fluoroquinolones, and macrolides [13,14].

### 2.1. Antibiotics and Immune Checkpoint Inhibitors in Lung Cancer

Despite previously unseen results in non-oncogene addicted NSCLC, only 20–40% of patients experience a durable benefit from immunotherapy [16], suggesting the importance of appropriately identifying those clinical, pathological, and/or laboratory parameters able to predict an optimized application of ICIs. Tumor-related factors alone, such as programmed death-ligand 1 (PD-L1) expression, tumor mutational burden, tumor-infiltrating lymphocytes, and high microsatellite instability, do not completely explain the existing differences in immunotherapy efficacy [17].

Considering this, many studies have focused on host factors. Among them, gut microbiota, clearly emerged as a crucial player in modulating the activity and toxicity of anticancer treatments, including immunotherapy [18]. In this regard, the development of an altered systemic immune response, mediated by gut microbiota dysbiosis [19], might contribute to explaining the reported worse prognosis observed with the concomitant administration of antibiotic therapy and ICIs [20].

Therefore, in the last few years, several studies and meta-analyses suggested the potential negative prognostic impact of antibiotics administration in immunotherapy-treated patients (Table 1).

The first meta-analysis that systematically evaluated the association between antibiotic therapy and clinical efficacy of ICIs in solid cancers was conducted in 2019 [21]. A statistically significant reduction in both overall survival (OS) and progression-free survival (PFS) was globally observed and confirmed for NSCLC in the subgroup analysis stratified by cancer type (OS: HR 2.68, 95% CI 2.19–3.28, *p* < 0.001; PFS: HR 1.79, 95% CI 1.29–2.49, *p* < 0.001). Regarding the time window of exposure, the survival impact of antibiotics when used within 60 days from immunotherapy initiation was weaker (HR 1.97, 95% CI 1.49–2.59, *p* < 0.001) if compared with their use within 30 days (HR 2.23, 95% CI 1.82–2.74, *p* < 0.001) [21].

In another meta-analysis of 18 observational studies [22], the timing of administration was categorized into three subgroups according to the results of a crucial study that showed a recovery time of 42 days for the human gut microbiota after antibiotic treatments [26]. A stronger effect on OS was reported when antibiotic therapy was applied in the 42 days before immunotherapy initiation, with a progressive decrease with later exposures. In detail, when comparing antibiotic-treated and untreated patients in the same timeframe, OS was 3.4 times longer in patients who did not receive antibiotics within 42 days before ICIs initiation (HR 3.43, 95% CI 2.29––5.14, *p* < 0.0001), 1.8 times longer for those unexposed to antibiotics within 60 days before and 42 days after the beginning of immunotherapy (HR 1.81, 95% CI 1.29–2.54, *p* = 0.001), while no differences were observed when considering antibiotics administration within 60 days before and anytime during ICIs treatment (HR 0.89, 95% CI 0.42–1.90, *p* = 0.76). A similar trend was described for PFS (group 1: HR 2.10, 95% CI 1.44–3.06, *p* < 0.001; group 2: HR 1.66, 95% CI 1.40–1.96, *p* < 0.001; group 3: HR 0.88, 95% CI 0.42–1.86, *p* = 0.75). Regarding cancer type, a statistically significant difference in outcome was confirmed for NSCLC patients (OS: HR 2.00, 95% CI 1.23–3.25, *p* = 0.0052; PFS: HR 1.64, 95% CI 1.07–2.52, *p* = 0.023) [22].

Similarly, a detrimental association between antibiotics and survival outcomes emerged from another meta-analysis. [23]. In the NSCLC cohort, a significant difference was reported in terms of median OS (7.83 months with antibiotics versus 17.19 without; HR 1.73, 95% CI 1.26–2.38, *p* = 0.0007) and PFS (2.28 versus 9.63 months; HR 1.39, 95% CI 1.16–1.67, *p* = 0.0004). Moreover, in line with the previously reported data, a higher death risk was observed for patients treated with antibiotics 1 month before or after ICIs initiation (HR 2.09, 95% CI 1.31–2.32, *p* = 0.002), becoming not significant when the time window was extended to 6 months before [23].

In a meta-analysis including only NSCLC patients, the HR was 1.47 for PFS (95% CI 1.13–1.90, *p* < 0.001) and 1.69 for OS (95% CI 1.25–2.29, *p* < 0.001), with a decrease of 1.2 months in median PFS and 6.7 months in median OS for patients treated with antibiotics [25]. The most detrimental effect was observed when antibiotics were administered between 60 days before and 60 days after ICIs initiation (OS: HR 2.04, 95% CI 1.49–2.79, *p* < 0.001; PFS: HR 1.72, 95% CI 1.30–2.27, *p* < 0.001) or within 60 days after (OS: HR 2.94, 95% CI 1.60–5.40, *p* < 0.001; PFS: HR 2.00, 95% CI 1.34–2.99, *p* < 0.001). Differently, no survival impact was documented for exposure to antibiotics within 90 days before, during the entire immunotherapy treatment, and at later times [25].

A worse OS (HR 1.80, 95% CI 1.28–2.55, *p* = 0.0008) and PFS (HR 1.70, 95% CI 1.27–2.27, *p* = 0.0004) were further confirmed in antibiotic-treated lung cancer patients, regardless of the histopathological type detected (in terms of the median proportion of squamous-cell carcinoma) [2]. Similarly, a worse prognostic impact was observed when antibiotics were administered within 60 days before ICIs initiation (OS: HR 2.58, 95% CI 1.94–3.34, *p* < 0.00001; PFS: HR 1.88, 95% CI 1.47–2.41, *p* < 0.00001) and from 60 days before to 60 days after (OS: HR 1.64, 95% CI 1.20, 2.24, *p* = 0.002; PFS: HR 2.01, 95% CI 1.55–2.61, *p* < 0.00001) in solid cancer patients [2].

Finally, the detrimental effect of antibiotic use in patients affected by solid tumors and treated with ICIs was further confirmed (OS: HR 2.07, 95% CI 1.51–2.84, *p* < 0.01; PFS: HR 1.53, 95% CI 1.22–1.93, *p* < 0.01) [24].

### 2.2. Antibiotics Timing and/or Cumulative Exposure: What Matters?

The time window of antibiotics exposure in relation to immunotherapy initiation seems to represent a crucial factor in determining the different outcomes of patients affected by NSCLC, as well as other solid tumors. While a statistically significant detrimental prognosis was consistently documented when antibiotics were administered shortly before and/or after ICIs initiation, this impact vanishes when the timeframe included immunotherapy course and later times (Supplementary Material).

In this sense, a monocentric analysis performed in cancer patients enrolled in ICIs phase I clinical trials reported a statistically significant negative effect on OS only in case of antibiotic use within 30 days prior to immunotherapy initiation (HR 2.00, 95% CI 1.2–3.3, *p* = 0.011) [27]. This finding was further confirmed in a prospective multicenter study, suggesting that prior antibiotic therapy (within 30 days before immunotherapy initiation), but not concurrent, was associated with worse ICIs activity and efficacy in patients treated in routine clinical practice (OS: HR 7.4, 95% CI 4.3–12.8, *p* < 0.001) [28]. In NSCLC patients, OS was 2.5 months in case of antibiotics administration within 30 days before ICIs therapy versus 26 months without any antibiotic exposure (*p* < 0.001) [28].

On the other hand, the detrimental prognostic effect of the antibiotic administration in ICIs-treated NSCLC patients might be associated with the antibiotic-immunotherapy exposure ratio (AIER), a candidate new variable defined as the proportion between days of antibiotic therapy and days of immunotherapy [3]. During the entire immunotherapy course, a higher AIER was reported to be related with a worse outcome, when considered as both a continuous variable (PFS: HR 1.053, *p* = 0.0029, OS: HR 1.064, *p* < 0.0001), as well as using the cutoff value of 4.2% (the median AIER in the whole immunotherapy treatment). In detail, when comparing patients with AIER < 4.2% and ≥4.2%, the median PFS was 3.5 versus 1.9 months (*p* < 0.0001) and the median OS was 13.2 versus 5.1 months (*p* = 0.0004). In contrast with the previously described meta-analyses, the antibiotic administration in the early ICIs period did not significantly influence patients’ prognosis [3]. Similarly, a retrospective analysis performed in ICIs-treated patients affected by advanced solid tumors, supporting the potential stronger prognostic impact of the cumulative antibiotic exposure variable compared with the administration timeframe [29].

### 2.3. Host Microbiome, Immunotherapy, and Antibiotics in Lung Cancer

The host microbiome, and gut microbiota in particular, has been widely recognized as a fundamental modulator of cancer development and progression, as well as of anticancer treatments’ efficacy and toxicity [30]. Among them, several chemotherapic drugs, such as 5-fluorouracil, gemcitabine, cyclophosphamide, irinotecan, cisplatin, and tyrosine kinase inhibitors, acting as targeted agents are included [31].

Regarding immunotherapy, commensal microorganisms might influence systemic immune functions and, therefore, treatment response through different mechanisms. A high diversity of the gut microbiome triggers Th1 lymphocyte and M1 macrophage differentiation, activation of helper and cytotoxic T cells, and upregulation of PD-1 expression [32]. In terms of composition, preclinical data showed that an overrepresentation of *Bifidobacterium* species in the gut microbiota of melanoma mouse models increased the response to anti-PD-L1 agents, with the possibility of restoring the antitumor activity through the oral administration of commensal *Bifidobacteria* in those mice harboring an unfavorable microbiota [33]. Moreover, the antitumor activity of cytotoxic T-lymphocyte antigen-4 (CTLA-4) inhibitors changed according to distinct *Bacteroides* species. Indeed, fecal transplantation with peculiar *Bacteroides* species (such as *B. fragilis*) from human to mice allowed remarkable responses to CTLA-4 blockade to be achieved [34]. The modification of gut microbiota with fecal transplantation or oral supplementation was also studied in a series of patients, including NSCLC, demonstrating that the clinical response to PD-1 blockade was correlated to the abundance in patients’ stool samples of *Akkermansia muciniphila*, probably potentiating the T lymphocytes-mediated immune response through an interleukin-12-dependent mechanism [35]. Focusing on NSCLC, responders to nivolumab had a higher gut microbiota diversity at treatment imitation, remaining stable during therapy [36].

In this light, antibiotics lead to an abnormal gut microbiota composition, potentially impairing ICIs efficacy, through different mechanisms. First, antibiotics administration might decrease the diversity of intestinal microbiota, eliminating the most immunogenic bacteria [37]. Second, early antibiotic use might influence the plasma levels of citrulline, a recognized biomarker of intestinal barrier and enterocytes integrity and functioning, that correlate with the clinical outcome in nivolumab-treated NSCLC patients [38]. Third, when commensal microbes decrease and pathogenic bacteria increase, toxins (such as the cytolethal distending toxin, the cytotoxic necrotizing factor-1, and the *B. fragilis* toxin), hydrogen sulfide, superoxide radicals and carcinogens, such as acetaldehyde, are abnormally released, potentially triggering DNA damages. Moreover, dysbiosis induces the lack of those anti-inflammatory actions mediated by the short-chain fatty acids normally fermented by the healthy microbiota, thus inducing an aberrant proliferation of the epithelial cells and, potentially, the carcinogenesis process [39]. Finally, another candidate mechanism is represented by the alteration of the β-catenin pathway, leading to loss of cell polarity, dysregulation of cellular growth, and acquisition of stem cell-like characteristics, due to the direct bond of microbial proteins to E-cadherin and/or to the translocation of some effectors into the host cells’ cytoplasm. Similar mechanisms are speculated to also be involved in the carcinogenesis process [40].

Although most of the available data in the oncological setting explored gut microbiota, a less-investigated but very promising microbial community is represented by the respiratory microbiota. The composition of lung microbiota is different from that characterizing other body districts and might be altered by smoking and environmental exposures [41]. Specifically, it seems to be determined by the balance of three main factors: microbial immigration, microbial elimination, and relative reproduction rates of its members [42]. Although specific communities are particularly enriched in lung cancer patients [43,44,45], the underlying mechanisms supporting this correlation are still far from being clarified. Nevertheless, the inflammatory response induced by quantitative and qualitative changes in lung microbiota is likely to be a determinant, as for other chronic respiratory diseases [46,47,48]. Interleukin-17C induced by aberrant bacteria (e.g., *Haemophilus influenzae*) increased the level of neutrophilic inflammation in COPD patients, thus mediating tumor proliferative effects [44]. Moreover, T helper cells induced by bacteria could promote angiogenesis and lung cancer cell proliferation [42]. In this light, epidemiological data suggested a correlation between prolonged antibiotic exposure and lung cancer development [49]. Finally, a complex bidirectional crosstalk between gut and lung microbiota has been demonstrated, in terms of both microbes transfer through bloodstream and lymphatic system and systemic immune response modulation. For example, short-chain fatty acids (SCFAs), metabolized by gut microbes and released in the systemic circulation, modulate several immune and epithelial cells functions through the regulation of G-protein-coupled receptors and histone deacetylase [50].

### 2.4. Open Issues and Future Perspectives in NSCLC Patients Treated with ICIs and Antibiotics

Although most of the available data are consistent in suggesting the negative prognostic impact of antibiotics administration in immunotherapy-treated patients, particularly if used shortly before and/or after ICIs initiation (Table S1 and Supplementary Material), other studies did not show this difference [3,51] or, even, they observed an opposite correlation [52,53].

Lack of definitive data in this setting might be related to different limitations. First, most of the available studies were retrospective, heterogenous in terms of patients’ selection, comorbidities, tumor burden, and treatment line. Second, the concurrent use of other drugs potentially modulating the immune response and host microbiome, such as corticosteroids and proton pump inhibitors (PPI), should be considered. In this regard, no detrimental effects were observed in terms of ICIs outcome when corticosteroids were administered as premedication, but their prognostic impact according to different dosages, timeframes, treatment durations, and administration routes is still debated [54]. Regarding PPI, in a pooled analysis of the OAK and POPLAR trials, *Chalabi* et al. reported a shorter PFS (1.9 versus 2.8 months; HR 1.30, 95% CI 1.10–1.53, *p* = 0.001) and OS (9.6 versus 14.5 months; HR 1.45, 95% CI 1.20–1.75, *p* = 0.0001) for PPI-treated versus not-treated NSCLC patients undergoing atezolizumab [55]. In addition, a strong heterogeneity was also observed in terms of antibiotic type, single or multiple antimicrobial drugs combinations, treatment durations, and administration routes. The retrospective study of *Mielgo-Rubio* et al. suggested that intravenous administration might be associated with a worse prognosis than oral (OS: 2.9 versus 14.2 months, *p* = 0.0001; PFS: 2.2 versus 5.9 months, *p* = 0.001) in advanced immunotherapy-treated NSCLC patients [56]. An additional crucial and still-unanswered question is whether the suspected detrimental effect on ICIs outcomes is really due to the antibiotics, rather than to the infection itself, as a direct cause or consequence of a systemic immune system impairment.

Finally, although microbiome dysbiosis is the most reliable mechanism justifying the harmful effect of antibiotics, a systematic evaluation of gut (and lung) microbiota composition and perturbation before, during, and after antibiotics and ICIs administration should be performed to provide concrete data. Moreover, when dealing with the microbiome, the existence of other potential modulators, such as the dietary pattern and the geographical origin, has to be considered within dedicated studies.

## 3. Viral Infections in Lung Cancer

Chronic viral infections represent frequent comorbidities in lung cancer patients. Furthermore, viral infections, such as HIV, HBV, and HCV, can increase the risk of developing malignancies, including lung cancer [57,58]. Specific antiviral therapies, such as antiretroviral therapy (ART) for HIV and antiviral drugs for HBV/HCV, are now the standard of care in chronic viral infections [59]. To date, most of the clinical trials testing immunotherapy in solid cancers have excluded patients affected by chronic viral infections, such as HIV, HBV, and HCV, due to concerns about viral reactivation, as well as ICIs safety and efficacy in these peculiar populations. Nowadays, dealing with viral infections, a specific mention should be made about the current Sars-Cov-2 pandemic that is spreading worldwide and features high aggressiveness and mortality in oncological patients, particularly in thoracic malignancies.

### 3.1. Human Immune-Deficiency Virus (HIV)

#### 3.1.1. HIV and Risk of Lung Cancer

Due to active antiretroviral therapy (ART), in the last years, the mortality and morbidity of PLWH has progressively decreased [60]. The HIV infection was historically associated with an increased risk of solid and hematologic cancers, including aggressive non-Hodgkin lymphoma, Kaposi sarcoma, cervical, and anal cancer, globally representing the acquired immune deficiency syndrome (AIDS)-defining cancers (ADCs) [61]. Nevertheless, due to the recent improved control of HIV infections, HIV-positive patients have a higher risk of developing other types of solid tumors, as lung cancer, gastrointestinal carcinoma, and skin cancer (known as non-AIDS-defining cancers, NADCs) [58]. Overall, cancer represents a leading cause of death for more than 37 million people worldwide living with HIV [62,63].

Regarding the risk of developing lung cancer, several factors might be implicated, such as the increased smoking habit in HIV-positive patients [64], the immunosenescence phenomenon due to their longer life expectancy [65], as well as the negative modulation of the immune system caused by the virus-related chronic inflammation [66]. Of interest, in the United States, HIV-positive patients, smokers, with a good disease control with ART are more likely to die for NSCLC than for AIDS [67].

#### 3.1.2. HIV and Immunotherapy in Lung Cancer

For several HIV-associated cancers, including lung cancer, treatment outcomes seem to be similar to those observed in the overall oncological population [68].

Regarding immunotherapy, although the use of ICIs might be theoretically beneficial for treating HIV-associated cancers, PLWH has usually been excluded from pivotal clinical trials testing these drugs, due to concerns about ICIs safety/efficacy and risk of viral reactivation.

**Figure 1:**
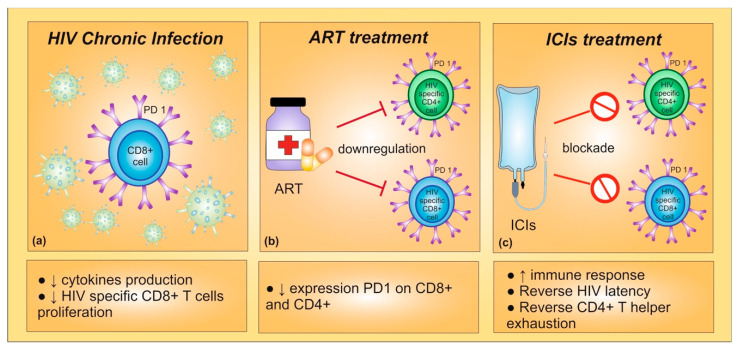
(**a**) During chronic HIV infection, high expression of PD-1 leads to a decrease in cytokines production and in HIV-specific CD8+ T cells proliferation. (**b**) Indeed, PD-1 expression is correlated with T cell exhaustion and disease progression in HIV-infected patients. The antiretroviral therapy (ART) seems to be able to inhibit PD-1 expression on CD8+ and CD4+ T cells. (**c**) The PD-1 blockade could provide an enhancement in CD8+ and CD4+ T cells and a restoration of the functional quality of virus-specific CD8+ T cells, improving immune response. Furthermore, immune checkpoint inhibitors could reverse HIV latency.

Preclinical data suggested that PD-1/PD-L1 blockade might induce an increased functioning of HIV-specific T cells. In detail, during chronic HIV infection, virus-specific CD8+ T cells might undergo a PD-1-mediated functional exhaustion, reducing proliferation and cytokine production. Indeed, PD-1 expression has been correlated with T cell exhaustion and disease progression in HIV-infected patients [69]. Furthermore, an effective ART has been shown to downregulate the expression of PD-1 on CD4+ and CD8+ T cells [70]. With these premises, PD-1 inhibition could play a crucial role in immune reconstitution of HIV-infected patients, highlighting the potential therapeutic benefit of blocking PD-1/PD-L1 interactions for enhancing CD8+ and CD4+ T cells [71]. In vivo studies confirmed that PD-1 blockade induced both an improvement in anticancer immune response and a restoration of the functional quality of virus-specific CD8+ T cells [69] (Figure 1).

Interestingly, the PD-L1 expression in NSCLC tissues was similar between HIV- and non-HIV-infected patients, but this biomarker was associated with poor prognosis particularly in patients with HIV infection, suggesting a more potent systemic immune suppression through the PD-1/PD-L1 axis in NSCLC patients with HIV than in those not infected [72].

A series of case reports [73,74], retrospective, and recent prospective studies (Table 2) investigated the safety and efficacy of ICIs in HIV patients affected by solid tumors, including lung cancer. In seven HIV-positive advanced NSCLC patients, nivolumab and pembrolizumab demonstrated a good safety profile with a similar efficacy compared to HIV-negative populations [75]. Similar results were obtained in another relatively small population of lung cancer patients [76]. Among 12 HIV-infected patients with advanced NSCLC treated with second-line nivolumab, a favorable clinical outcome (7/12 disease control with 3 partial responses and 4 disease stabilizations), without clinically meaningful side effects (except one case of neurosyphilis) and neither a relevant impact on HIV viral load nor CD4+/CD8+ cell count were reported. Furthermore, nivolumab was able to enhance the capacities of HIV-specific CD8+ cells to proliferate and secrete cytokines, expanding the PD-1 low T cell subset [77].

An observational French study by the CANCERVIH network, evaluated 23 HIV-positive cancer patients (including 21 advanced NSCLC) treated with nivolumab or pembrolizumab, reporting a good tolerability, a disease control rate (DCR) of 41% and a median OS of 10.7 months. Of note, no significant impact on CD4+ count or HIV viremia was described [78].

In another retrospective analysis, including NSCLC as the predominant tumor type in the HIV cohort (*n* = 12), the toxicity and efficacy rates were similar to those observed in patients without chronic viral infections. In detail, the objective response rate (ORR) was 13% among 8 NSCLC patients treated with ICIs monotherapy and 75% among 4 NSCLC patients treated with chemo-immunotherapy combination. Again, viral reactivations were not observed [84].

A dedicated phase I clinical trial evaluated as the primary endpoint the safety of pembrolizumab in 30 PLWH, 11 with ADCs and 19 with NADCs, including one NSCLC patient (sarcomatoid carcinoma). The most common immune-related adverse events were fatigue, hypothyroidism, and anemia. HIV was controlled in all participants, without any statistically significant increase in CD4+ cells count. In addition, promising signals of activity were observed among patients affected by Kaposi sarcoma, lung cancer, primary effusion lymphoma, and diffuse large B-cell lymphoma. Of note, the NSCLC patient obtained a complete objective response due to pembrolizumab treatment [4].

In order to summarize the safety and efficacy results of ICIs in HIV-positive patients with advanced-stage cancers, a systematic review was performed [85]. The included immunotherapy agents were nivolumab, pembrolizumab, ipilimumab, and nivolumab plus ipilimumab. NSCLC was the most prevalent cancer type (34.2%), followed by melanoma (21.9%) and Kaposi sarcoma (12.3%). Overall, 95% of patients were receiving ART at the time of immunotherapy initiation (mainly in monotherapy, but 5 patients received them in combination with anti-PD-1/anti-CTLA-4 inhibitors). Among 37 patients with a known baseline viral load, 31 (83.8%) had an undetectable viral load. In terms of safety, ICIs were generally well tolerated. None of the included studies reported the occurrence of immune reactivation inflammatory syndrome. Of note, three patients had chronic hepatitis infection (two HCV, one HBV), none of whom showed any treatment-related change in liver function. Among 34 patients with known paired pre- and post-treatment HIV loads, HIV remained suppressed in 93%, with undetectable HIV load. Among 25 patients with paired pre- and post-treatment CD4+ cells values, the count significantly increased with a mean change of 12.3/μL. In terms of antitumor activity, ORR was overall promising: 30% for NSCLC, 27% for melanoma, and 63% for Kaposi sarcoma [85].

The Checkmate 817 trial is a phase 3b/4 study that aimed to evaluate the efficacy and safety of the nivolumab plus ipilimumab combination in metastatic previously untreated NSCLC patients, including a special cohort of ECOG Performance Status (PS) 2 or ECOG PS 0-1 and 1 among: asymptomatic untreated brain metastases, hepatic or renal impairment, HIV. Four HIV-positive patients (7%) were enrolled. In 2019, the first results of this trial showed a consistent safety profile and promising OS outcomes in the special advanced NSCLC population [79].

Recently, results from the DURVAST trial, a phase 2 study evaluating durvalumab in HIV-infected patients with solid tumors, were published. Among 20 enrolled patients, 14 had NSCLC, 2 melanoma, 1 small-cell lung cancer (SCLC), 2 anal carcinoma, and 1 bladder carcinoma. All patients, receiving ART, showed undetectable HIV plasma viremia. Durvalumab demonstrated to be feasible (the primary endpoint, defined as the ability to receive at least a median number of 4 cycles) and safe. The most common adverse events were diarrhea, arthromyalgia, and asthenia. There were no safety concerns related to HIV reactivation, HIV viremia remained undetectable, and the CD4+/CD8+ T cells count was stable throughout durvalumab treatment. Of note, 1 NSCLC patient with a CD4+ T-cell count of less than 200 cells/mm^3^ had no side effects and experienced a long-lasting partial response, suggesting that treating patients with low basal CD4+ T cell counts might also be safe [80]. In terms of antitumor activity, DCR, evaluated in 16 patients, was 50%, including long-lasting responses. The results of this trial, although limited by the small sample size, suggested a longer duration of clinical benefit in patients treated with integrase strand-transfer inhibitors (INSTIs), a newer class of antiretroviral drugs, allowing the authors to speculate that INSTIs could contribute to the antitumoral immune response of durvalumab [80].

The results of a phase 1 trial of nivolumab in advanced HIV-associated solid tumors, including NSCLC and SCLC, were recently presented [81]. The primary endpoint was the safety and feasibility of nivolumab at standard doses; the secondary endpoints were the evaluation of nivolumab impact on immune function (HIV viral load, CD4+/CD8+ cells) and objective response. A total of 11% of patients experienced treatment-related serious adverse events, mainly fatigue and rush. An ORR of 24% was observed in immunotherapy-responsive cancers. Moreover, there were no significant changes in HIV viral load during the study period. In light of these findings, the authors concluded that HIV-infected patients treated with ART, having a CD4+ T-cell count greater than 100 cells/μL and undetectable viral load, might safely receive nivolumab in clinical trials.

The French Cooperative Thoracic Intergroup reported the preliminary results of the CHIVA-2 trial, a phase II study of nivolumab after prior chemotherapy for HIV-infected advanced NSCLC patients [82]. A total of 16 patients were enrolled. The majority of them (69%) received nivolumab as second-line treatment, with a median treatment duration of 3.5 months. Similarly to what was observed in the general NSCLC population, a DCR (primary endpoint) of 62.5%, with a median PFS and OS (secondary endpoints) of 3.4 and 14.1 months, respectively, were reported. Nivolumab was well tolerated without serious adverse events nor opportunist infections reported [82].

Of interest, recently designed trials, such as the phase 3 EMPOWER-Lung 1 trial with cemiplimab monotherapy, opened the enrolment to patients with controlled hepatitis B or C, or HIV [83].

Finally, another early phase trial is currently ongoing in order to specifically evaluate the efficacy and safety of nivolumab in combination with cabozantinib (NCT04514484) in HIV-positive patients, including lung cancer [Table 2].

### 3.2. HBV/HCV and Immunotherapy in Lung Cancer

Only limited data are currently available about the safety and efficacy of immunotherapy in lung cancer patients with past or chronic HBV or HCV infection, mainly due to their exclusion from pivotal clinical trials, as described for the HIV-positive population.

Among seven patients treated with nivolumab or pembrolizumab for metastatic melanoma (*n* = 2) or NSCLC (*n* = 5), affected by a chronic or past HBV/HCV infection, only one patient affected by metastatic melanoma showed an increase in alanine aminotransferase (ALT) of grade 2 according to the Common Terminology Criteria for Adverse Events (CTCAE). Only grade 1 ALT increases or no hepatic toxicities at all were reported in the remaining patients. Efficacy results were similar to those expected in patients without viral hepatitis [86].

A retrospective analysis focused on immunotherapy in special populations, such as patients with organ transplant, HIV-positive, or with HBV/HCV infection, included only one HCV-infected patient undergoing anti-viral treatment (ledipasvir and sofosbuvir) and affected by an advanced NSCLC treated with nivolumab. This patient showed an undetectable viral load, without experiencing any toxicity [87].

In a retrospective study, 34 HBV/HCV-infected patients were included, most of them affected by hepatocellular carcinoma (*n* = 17) and lung cancer (*n* = 11). Overall, any grade of immune-related adverse events was observed in 44% of patients, with grade ≥3 29%. The ORR was 21%. Among six patients with known pre/post-treatment viral titers (2 HCV and 4 HBV), no evidence of viral reactivation was observed [79].

The largest retrospective series of advanced immunotherapy-treated NSCLC patients with concurrent HBV/HCV infection included 19 patients (16 with past or chronic HBV, two of them with HCV co-infection, and five patients with chronic HCV infection). The administration of ICIs in this population appeared to be safe. In detail, no severe hepatic immune-related adverse events were reported; moreover, the baseline liver function test abnormalities or the presence of an active viral infection were not predictive of a worse liver function during treatment. The ORR was 35%, with a median PFS of 4.5 months, including deep and prolonged responses among several patients [5].

### 3.3. Open Issues and Future Perspectives in HIV, HBV, and HCV Lung Cancer Patients Treated with ICIs

The above-mentioned studies consistently supported that lung cancer patients with chronic viral infections, such as HIV, HBV, and HCV, are likely to achieve a clinically meaningful benefit from immunotherapy, comparable to that reported in not-infected oncological patients, without unexpected toxicities.

Antiretroviral therapy in PLWH has dramatically reduced HIV-related morbidity and mortality. HIV-positive patients treated with ART, experiencing a good infection control in the absence of clinical and/or laboratory findings, in particular with preserved CD4+ function, should be considered as potential candidates for the enrollment in clinical trials with immunotherapy [68]. Of note, concurrent ICIs and ART have not led to a dramatic change in CD4+ counts in most of the patients, which suggests that immune checkpoint blockade does not impair HIV management and may improve CD4+ counts in selected patients [88].

A multidisciplinary strategy based on a close collaboration between the medical oncologist and the infectious disease specialist seems to be crucial for lung cancer patients and concomitant viral infections.

Regarding HIV, those patients affected by a poorly controlled infection and/or AIDS should be carefully evaluated for the potential risk of inflammatory symptoms, systemic viremias, or immune-related pneumonia. An algorithm for the evaluation of PLWH who are under consideration for ICIs initiation was recently proposed, based on the availability of clinical trials and on the HIV status (CD4+ count and viral load). In this algorithm, a crucial condition for ICIs eligibility, both in standard practice and in clinical trials, is the presence of a well-controlled HIV infection [89].

Despite the very limited data available for lung cancer patients with concurrent HBV/HCV infection, no safety concerns emerged related to immunotherapy, which maintains a promising efficacy. In this setting, the integrated multidisciplinary approach should include a hepatologist, together with a medical oncologist and an infectious disease specialist, in order to consider association with specific antiviral therapy, periodical viral load monitoring, and early interception of those rare cases of relevant hepatotoxicity.

Some ongoing studies, such as the DURVAST and CHIVA2 trials, will help to clarify whether immunotherapy for these special subgroups of lung cancer patients could be safely used, maintaining the well-known efficacy observed in patients without chronic infections.

## 4. Sars-Cov-2 Infection and Lung Cancer

Since the end of 2019, the COVID-19 pandemic is progressively spreading worldwide, causing more than 49,100,000 cases and 1,239,157 deaths [6]. Although we should consider that the extremely high COVID-19-related pressure on the healthcare systems might lead to confounding findings, a growing amount of data suggests that oncological patients are particularly vulnerable in terms of risk of both severe events (hospitalization up to 40%, severe respiratory illness 20%) and death (13–35%) compared with patients without a history of cancer [7,8,9,90]. Beyond patient-related parameters, such as age, sex, smoking status, and comorbidities, some cancer-specific factors have been associated with an increased mortality, mainly the PS according to ECOG and the presence of an active oncological disease [90].

Thoracic cancers are at a higher risk of death compared to the general population but also to other cancer types, with an estimated mortality rate of around 32% [10]. Among patients affected by thoracic malignancies, older age (>65 years), male sex, former or active smoking status, prior steroids, and PS according to ECOG >2 emerged as crucial variables related to COVID-19 mortality [91].

Although some studies did not identify a significant association between recent treatments and COVID-19 outcome [7,9,90], the real correlation between the type and timing of anticancer therapies, including immunotherapy, and COVID-19 complications and mortality still needs clarification.

Regarding immunotherapy, some reports initially described the occurrence of explosive deteriorations of the patient’s clinical condition related to the concomitant ICIs administration and SARS-CoV-2 infection, probably due to a pathological hyper-activation of CD8+ T-cells, inducing an aberrantly excessive immune response (cytokine-storm), finally leading to the severe acute respiratory distress syndrome [92]. Nevertheless, although the occurrence of a negative synergy cannot always be excluded, other evidence did not find a significant association between prior PD-1 blockade administration (at least in monotherapy) and COVID-19 outcomes [93]. Similarly, in the TERAVOLT study, immunotherapy alone and tyrosine-kinase inhibitors were not associated with an increased risk of death, differently from chemotherapy, both as unique modality or in combination with ICIs [91]. A recent update of the COVID 19 and Cancer Consortium (CCC19) analysis on >3000 patients suggested that the 30-day mortality was higher among those undergoing an active treatment (with the exception of endocrine therapy), particularly within 1–3 months prior to COVID-19 diagnosis. Of interest, 30-day mortality was the highest for patients treated with chemoimmunotherapy [94].

### Open Issues and Future Perspectives of Sars-Cov-2 Infection in Lung Cancer Patients

The Sars-Cov-2 infection translated into a previously unseen challenge for all cancer patients, but particularly for those affected by thoracic malignancies, for whome higher risk of mortality has been largely confirmed [10]. Although immunotherapy for lung cancer patients led to clinically relevant improvements in both prognosis and quality of life, the current Sars-Cov-2 pandemic poses a series of concerns related to ICIs administration.

Beyond safety issues, mainly related to the combination of immunotherapy with cytotoxic chemotherapy, diagnostic difficulties might emerge in discriminating COVID-19 from disease-related symptoms, lung cancer progression, as well as from immune-related adverse events [95].

Although these challenges, it should be underlined that COVID-19 infection represents only a small proportion of the deaths occurring in lung cancer patients, confirming the urgent need of prioritizing anticancer treatment even during this pandemic, as stressed by guidelines and expert opinions worldwide [96]. Nevertheless, type, timing, and duration of treatment but mainly patient’s selection criteria should be carefully considered in order to optimize lung cancer care during the Sars-Cov-2 outbreak.

Finally, COVID-19 clinical trials specifically dedicated or at least opened to cancer patients should be advocated for to identify appropriate strategies to treat and hopefully prevent (vaccination) the Sars-Cov-2 infection, tailored to the oncological setting.

## 5. Conclusions

The concomitant diagnosis of bacterial or viral infections in patients affected by lung cancer represents a relevant issue in clinical practice, particularly nowadays that the main therapeutical strategy includes immunotherapy and, therefore, the modulation of the systemic immune response.

Although bacterial and viral infections are very common, many underlying mechanisms are still unclear in these special populations. Dedicated prospective studies are urgently needed to clarify the correlation between antibiotics type, route and duration of administration, type of bacterial infections, concurrent use of other drugs, and ICIs efficacy. Moreover, the role of gut microbiota in response to ICIs and the crosstalk with lung microbiota should be explored in order to prevent wrong use of antibiotics, as well as to identify potential therapeutical approaches exploiting this crosstalk.

Although patients affected by chronic viral infections were excluded from clinical trials, many studies supported the efficacy and safety of immunotherapy for infected NSCLC patients, in a similar way with the general oncological population. A multidisciplinary approach is required for the optimal clinical management of these patients.

Nowadays, the Sars-Cov-2 outbreak represents a previously unseen challenge for cancer patients, especially for those affected by thoracic malignancies, for the increased complications and mortality risk they might experience, as well as for the diagnostic-therapeutical issues that have clearly emerged. International recommendations should be applied to ensure lung cancer patients have the best care even in this emergency situation.

## Figures and Tables

**Table 1:** Meta-analyses evaluating the impact of antibiotics administration on clinical outcomes of advanced cancer patients treated with immune-checkpoint inhibitors, focusing on results obtained in non-small-cell lung cancer.

Meta-Analysis.	*N*. of Included Studies	Pooled HR PFS [95% CI] *p*-Value	Pooled HR OS [95% CI] *p*-Value	NSCLC–HR PFS [95% CI] *p*-Value	NSCLC–HR OS [95% CI] *p*-Value
[21]	19	1.84 [1.49–2.26] *p* < 0.001	2.37 [2.05–2.75] *p* < 0.001	1.79 [1.29–2.49] *p* < 0.001	2.68 [2.19–3.28] *p* < 0.001
[22]	18	1.65 [1.3–2.1] *p* < 0.0001	1.92 [1.37–2.68] *p* < 0.001	1.64 [1.07–2.52] *p* = 0.0023	2.00 [1.23–3.24] *p* = 0.0052
[23]	20	1.53 [1.30–1.79] *p* < 0.01	1.90 [1.55–2.34] *p* < 0.01	1.39 [1.16–1.67] *p* = 0.0004	1.73 [1.26–2.38] *p* = 0.0007
[24]	15	1.53 [1.22–1.93] *p* < 0.01	2.07 [1.51–2.84] *p* < 0.01	N.A.	N.A.
[25] *	23	N.A.	N.A.	1.47 [1.13–1.90] *p* < 0.01	1.69 [1.25–2.29] *p* < 0.01
[2]	33	1.76 [1.47–2.12] *p* < 0.00001	1.76 [1.41–2.19] *p* < 0.00001	1.70 [1.21–2.27] *p* = 0.0004	1.80 [1.28–2.55] *p* = 0.0008

HR, hazard ratio; OS, overall survival; PFS, progression-free survival; NSCLC, non-small-cell lung cancer; N.A., not available. * Only NSCLC patients were included.

**Table 2:** Clinical studies investigating safety and efficacy of ICIs in HIV patients affected by solid tumors, including lung cancer.

Trial	Phase	Type of Cancer	N. of Patients	Type of ICIs	Primary Endpoints	Secondary Endpoints	Main Findings	Status
[4] [NCT02595866]	1	Advanced or metastatic solid and hematological cancers	60 HIV-positive *	Pembrolizumab	Safety	ORR, PFS, DoR, OS	Results on 30 patients: - most common irAEs: fatigue, hypothyroidism, anemia; - signals of activity in several cancer types; - the only one NSCLC patient obtained CR.	Recruiting
[79] [NCT02869789]	3b/4	Advanced or metastatic NSCLC	1036 [4 HIV-positive in cohort 1A] §	Nivolumab plus Ipilimumab	Safety	PFS, ORR, DoR, FACT-L, OS	- good safety profile; - promising OS outcomes.	Active, not recruiting
[80] [NCT03094286]	2	Advanced or metastatic solid and hematological cancers	20 HIV-positive [14 NSCLC, 2 melanoma, 1 SCLC, 2 anal carcinoma, 1 bladder carcinoma] §	Durvalumab	Feasibility (ability to receive at least a median number of 4 cycles)	ORR, PFS, OS	- undetectable HIV viremia in all patients; - durvalumab was feasible and safe; - DCR (on 16 patients) 50%; - one NSCLC patient with CD4+ cell count<200 had no side effect with long-lasting PR.	Active, not recruiting
[81] [NCT02408861]	1	Advanced or metastatic solid and hematological cancers	96 HIV-positive *	Nivolumab plus Ipilimumab	MTD	ORR, immune function, change in immune status, change in HIV viral load	Results on 37 patients: - 11% of patients showed irAEs; - ORR 24% in immunotherapy responsive; - no changes in HIV viremia.	Recruiting
[82] [NCT03304093]	2	Advanced or metastatic NSCLC	16 HIV-positive §	Nivolumab	DCR	PFS, OS, tolerance, impact on HIV control, DoR	- DCR of 62.5%; - median PFS and OS of 3.4 and 14.1 months; - no serious adverse events or opportunist infections reported.	Active, not recruiting
[83] [NCT03088540]	3	Advanced or metastatic NSCLC	712 [not yet specify the number of HIV-positive patients] §	Cemiplimab vs. SOC	OS, PFS	ORR, DOR, BOR	Not yet available data on HIV population	Active, not recruiting
NCT04514484	1	Advanced or metastatic solid cancers	18 HIV-positive *	Nivolumab plus Cabozantinib	Safety and feasibility (ability to receive at least a median number of 4 cycles)	DOR, PFS, OS, analysis on HIV reservoir	NA	Recruiting

ICIs, immune checkpoint inhibitors; NSCLC, non-small cell lung cancer; HCC, hepatocellular carcinoma; ORR, objective response rate; PFS, progression-free survival; DoR, duration of response; OS, overall survival; irAEs, immune related adverse events; CR, complete response; FACT-L, functional assessment of cancer therapy-lung; SCLC, small-cell lung cancer; DCR, disease control rate; PR, partial response; MTD, maximum tolerated dose; SOC, standard of care; BOR, best overall response; NA, not available. * Estimated enrollment, § actual enrollment.

## Data Availability

Data set available on request to corresponding authors.

## Supplementary Materials

The following are available online at https://www.mdpi.com/1422-0067/22/1/42/s1, Table S1: Impact of antibiotics administration on immunotherapy clinical outcomes according to the time window exposure to antibiotics (only meta-analyses were included).
