# Supplementary material for: Infections and Immunotherapy in Lung Cancer: A Bad Relationship?
Source: Int J Mol Sci. 2020 Dec 22;22(1):42. doi: 10.3390/ijms22010042 (PMC7793072; doi:10.3390/ijms22010042)
Supplement: Supplementary file 1 [file ijms-22-00042-s001.pdf]

International Journal of
Molecular Sciences

MDPI

Supplementary Material

Table 1. Impact of antibiotics administration on immunotherapy clinical outcomes according to the time window exposure to antibiotics (only meta-analyses were included).

Meta-analysis	Time Window Exposure to ATX in Relation to ICIs	HR PFS [95% CI] *p*-value	HR OS [95% CI] *p*-value
Huang et al., 2019	Anytime before ICIs initiation —ICIs initiation	1.70 [1.43–2.02] *p* < 0.001	2.29 [1.92–2.73] *p* < 0.001
	One month before ICIs initiation—ICIs initiation	N.A.	2.23 [1.82–2.74] *p* < 0.001
	Two months before ICIs initiation—ICIs initiation	N.A.	1.97 [1.49–2.59] *p* < 0.001
	Anytime before or after ICIs initiation	1.91 [1.31–2.78] *p* = 0.001	2.56 [1.96–3.36] *p* < 0.001
Wilson et al., 2020	42 days before ICIs initiation —ICIs initiation	2.10 [1.44–3.06] *p* < 0.001	3.43 [2.29–5.14] *p* < 0.0001
	60 days before—42 days after ICIs initiation	1.66 [1.4–1.96] *p* < 0.001	1.81 [1.29–2.54] *p* = 0.001
	60 days before—anytime during ICIs	0.88 [0.42–1.86] *p* < 0.75	0.89 [0.42–1.90] *p* = 0.76
Xu et al., 2020	Six months before ICIs initiation	N.A.	1.81 [0.91–3.63] *p* = 0.09
	One month before or after ICIs initiation	N.A.	2.09 [1.31–3.32] *p* = 0.002
Petrelli et al., 2020	Two months before ICIs initiation—ICIs initiation	N.A.	2.33 [1.33–3.34] *p* < 0.01
	Three months after ICIs initiation	N.A.	Not significant (only 1 study)
Lurienne et al., 2020	90 days before ICIs initiation—ICIs initiation	1.56 [0.78–3.13] *p* = 0.21	2.49 [0.95–6.51] *p* = 0.063
	60 days before—60 days after ICIs initiation	1.72 [1.30–2.27] *p* < 0.001	2.04 [1.49–2.79] *p* < 0.001
	60 days after ICIs initiation	2.00 [1.34–2.99] *p* < 0.001	2.94 [1.60–5.40] *p* < 0.001
	90 days before or after ICIs initiation	0.97 [0.44–2.17] *p* = 0.95	1.24 [0.56–2.76] *p* = 0.61
Yang et al., 2020	60 days before ICIs initiation—ICIs initiation	1.88 [1.47–2.41] *p* < 0.00001	2.58 [1.94–3.43] *p* < 0.00001
	60 days before—60 days after ICIs initiation	2.01 [1.55–2.61] *p* < 0.00001	1.64 [1.20–2.24] *p* = 0.002
	60 days before and anytime during ICIs initiation	1.29 [0.77–2.17] *p* = 0.33	1.38 [0.89–2.15] *p* = 0.15

ATX, antibiotics; HR, hazard ratio; PFS, progression-free survival; OS, overall survival; ICIs, immune checkpoint inhibitors, N.A., not available.
